# From Bacterial Diversity to Zoonotic Risk: Characterization of Snake-Associated *Salmonella* Isolated in Poland with a Focus on Rare O-Ag of LPS, Antimicrobial Resistance and Survival in Human Serum

**DOI:** 10.3390/ijms262412018

**Published:** 2025-12-13

**Authors:** Michał Małaszczuk, Aleksandra Pawlak, Stanisław Bury, Aleksandra Kolanek, Klaudia Błach, Bartłomiej Zając, Anna Wzorek, Gabriela Cieniuch-Speruda, Agnieszka Korzeniowska-Kowal, Andrzej Gamian, Gabriela Bugla-Płoskońska

**Affiliations:** 1 Department of Microbiology, Faculty of Medicine, Wroclaw Medical University, 50-368 Wroclaw, Poland; michal.malaszczuk@umw.edu.pl; 2 Department of Microbiology, Faculty of Biological Sciences, University of Wrocław, 50-137 Wroclaw, Poland; 3 Department of Comparative Anatomy, Institute of Zoology and Biomedical Research, Faculty of Biology, Jagiellonian University, 30-387 Krakow, Poland; 4 NATRIX Herpetological Association, 52-010 Wroclaw, Poland; 5 Department of Geoinformatics and Cartography, Institute of Geography and Regional Development, Faculty of Earth Sciences and Environmental Management, University of Wrocław, 50-137 Wroclaw, Poland; 6 Department of Laboratory Diagnostics, University Clinical Hospital, Borowska 213, 50-556 Wroclaw, Poland; 7 Department of Immunology of Infectious Diseases, Hirszfeld Institute of Immunology and Experimental Therapy, Polish Academy of Sciences, 53-114 Wroclaw, Poland

**Keywords:** *Salmonella*, One Health, zoonosis, human serum resistance, antimicrobial resistance, LPS

## Abstract

The One Health approach emphasizes the importance of zoonoses due to their pandemic potential, highlighting the need to characterize emerging bacterial pathogens across animal reservoirs. Non-typhoidal *Salmonella* (NTS) species are among the most common zoonotic agents and can be transmitted by various reservoirs, including reptiles. Both direct and indirect contact with reptiles may result in Reptile-Associated Salmonellosis (RAS), which mainly affects children, immunocompromised individuals, pregnant women, and the elderly. This study aimed to isolate and characterize the Gram-negative intestinal microbiota from free-living snakes in Poland (*Natrix natrix, Natrix tessellata, Coronella austriaca, Zamenis longissimus,* and *Elaphe dione*) and to determine the prevalence and virulence potential of *Salmonella*. Using MALDI-TOF Mass Spectrometry, 432 isolates were identified. Serological analysis of 62 *Salmonella* isolates revealed 10 distinct O-antigen groups, and rare serovars O:38, O:48, O:57 and others were confirmed. *Salmonella* isolates were tested for antibiotic susceptibility and resistance to Human Serum; most isolates survived exposure to serum while remaining susceptible to antibiotics. One isolate was classified as multidrug-resistant (MDR), showing resistance to amoxicillin/clavulanic acid, ampicillin, cefuroxime, cephalexin, tigecycline, and fosfomycin. These findings demonstrate that wild snakes in Poland can act as reservoirs of pathogenic and zoonotic *Salmonella*, emphasizing their epidemiological significance in natural ecosystems.

## 1. Introduction

One Health is a multidisciplinary approach that aims to sustainably balance and optimize the health of people, animals and ecosystems. It focuses on human infectious diseases, zoonoses, multidrug resistance, foodborne diseases, and biodiversity [1]. It prioritizes zoonoses as diseases of greatest concern because of their pandemic potential, as humanity has painfully learned during pandemics such as COVID-19, H1N1 avian influenza, the Ebola epidemic or foodborne outbreaks of *Escherichia coli*. Zoonoses constitute 75% of all emerging diseases discovered in the last 20 years, and their transmission is affected by global environmental changes and increasing human pressure on natural ecosystems. The temperature rise caused by global change is affecting entire ecosystems, which in turn leads to changes in the transmission of pathogens among animals, plants, and humans [2]. Therefore, it is crucial to expand knowledge about zoonotic pathogens in order to control them and to identify opportunities to prevent further outbreaks or reduce their negative effects on humans, animals and ecosystems. For that reason, the European Union imposes on member countries the obligation to monitor and report zoonoses under Directive 2003/99/EC. According to it, member countries are obliged to provide information on zoonoses, with a focus on zoonotic agents, drug resistance of pathogens and foodborne outbreaks (FBOs). The 8 most concerning zoonotic agents are specified in the List A Annex of the above-mentioned directive and are as follows: *Salmonella*, *Campylobacter*, *Listeria monocytogenes*, Shiga toxin-producing *E. coli* (STEC), *Mycobacterium bovis*, *Brucella*, *Trichinella* and *Echinococcus* [3]. As expected, according to The European Union One Health 2023 Zoonoses Report, the number of zoonoses in the EU is increasing [4]. Among zoonotic agents mentioned above in the EU, *Salmonella* rods are one of the most common—in 2023 there were 77,486 cases (an increase of 16.9% compared to 2022) recorded, followed by 148,181 cases (an increase of 4.3% compared to 2022) due to campylobacteriosis and 10,217 cases (an increase of 30.0% compared to 2022) due to STEC infections. *Salmonella* can be transmitted by a variety of reservoirs, humans, animals, plants and whole ecosystems, but most reports mainly monitor only poultry and eggs for the presence of these bacteria, since these are the most common sources of salmonellosis associated with the food chain [5]. However, salmonellosis is increasingly reported as a result of eating vegetables and fruit contaminated by animal microbiota. It is noticeable that in recent years, people are increasingly changing their eating habits, eating more vegetables and fruits and limiting processed and meat products. It is worth noting that for most food pathogens, including *Salmonella*, thermal processing of contaminated food is a factor limiting infection [6]. However, most vegetables and fruits are eaten raw, which carries a high risk of foodborne infections [7]. Given this, in the case of salmonellosis, monitoring of critical points in the food chain should be expanded. Particularly in the production of prepackaged leafy green vegetables, contamination by small wild vertebrates found in ready-to-eat packages in supermarkets is becoming increasingly common [8]. What is important in this context is the fact that, ectotherms animals, mainly reptiles and amphibians, are an important reservoir of *Salmonella* spp. Scientific literature indicates that *Salmonella* rods are often a component of the natural intestinal microbiota of these animals, without causing any disease symptoms [9]. However, many factors such as stress, change in food, presence of parasites, bacterial or viral infections can lead to the manifestation of symptoms of salmonellosis in reptiles and amphibians [10,11,12,13,14]. Importantly, both direct and indirect contact with reptiles can lead to infections in humans that have been described since the 1970s as Reptile Associated Salmonellosis (RAS). It mainly concerns small children, people with immune deficiencies, pregnant women and the elderly. What is important, RAS are more serious than other non-typhoidal salmonellosis (NTS) and may lead to sepsis and systemic infections, which are potentially fatal. Therefore, in some countries, such as the USA and China, RAS monitoring is carried out. Mean, while in European countries there are no such regulations, that’s why we pay attention to the need for change. The first case of RAS was documented in 1940s, and outbreaks of RAS have been regularly reported since then. Knowledge about the virulence factors of *Salmonella* is increasing, but still full of gaps, particularly in terms of RAS. *Salmonella* serovars can be divided according to mammalian and bird hosts specificity into: broad-host-range or generalist, host-adapted, and host-restricted serovars. The interesting fact is that the host-restricted and host-adapted serovars relate to systemic infections much more often than to gastroenteritis. It relates to changes in the *Salmonella* virulence plasmid (pSV), accumulation of pseudogenes and chromosome rearrangements [13]. In the literature review, most *Salmonella* serovars isolated from reptiles belong to subspecies: I (70.3%), IIIb (29.7%) and II (19.6%). Among free-living reptiles, *Salmonella* spp. are most often isolated from snakes [15]. The data varies depending on many factors, e.g., geographical region, ambient temperature, research methods, and the year of obtaining the research results. It should be emphasized that reptiles, especially snakes, are among the most difficult vertebrates to study—they are secretive, usually occur in low densities and are difficult to find and catch. Additionally, global changes, human impact on the natural environment, changes in eating habits, and increased migration of animals and people can have a huge impact on spread and severity of RAS. Most salmonellosis in humans occurs in summer. The change in room temperature caused by global warming accelerates the growth of *Salmonella*. An average increase of 1 degree Celsius in the maximum weekly ambient temperature causes an increase in the weekly number of salmonellosis by 8.8% [16]. Therefore, it is necessary to assess current prevalence and virulence factors of *Salmonella* and cohabiting microbiota to get insight into potential risk factors of future RAS outbreaks. Here, we employed a multidisciplinary approach to intestinal infections, including RAS. Obtaining new data from different regions of the EU and combining them with data from other areas in the context of the One Health approach may contribute to the implementation of better methods of monitoring and preventing salmonellosis.

The aim of this study was to isolate and characterize Gram-negative intestinal microbiota from free-living snakes in Poland, with particular emphasis on determining the prevalence and virulence traits of *Salmonella* serovars. Snakes are widespread across various habitats and play an important ecological role as potential vectors of zoonotic microorganisms. Understanding their microbiota is therefore essential for evaluating the circulation of pathogens in natural ecosystems. Our results concern four free-living snake species—*Coronella austriaca, Zamenis longissimus*, *Natrix natrix* and *Natrix tessellata,* and two kept species—*Nerodia fasciata* and *Elaphe dione* for which no data, or limited data, on intestinal microbiota composition or *Salmonella* occurrence have been reported. Additionally, the study examined antibiotic resistance, reflecting environmental selective pressure, and susceptibility to the human complement system, which indicates the potential ability of these bacteria to survive and proliferate in human serum during systemic infection.

## 2. Results

### 2.1. Sample Collections

A total of 78 swab samples were collected from wild snakes, from which 420 bacterial isolates were obtained. Of these, 156 isolates (37%) originated from *N. natrix* (NN), 114 (27%) from *C. austriaca* (CA), 95 (23%) from *N. tessellata* (NT), and 55 (13%) from *Z. longissimus* (ZL). Additionally, 12 bacterial isolates were obtained from three swabs collected from breeding individuals of *E. dione*.

### 2.2. Matrix-Assisted Laser Desorption/Ionization—Time of Flight Mass Spectrometry Identification (MALDI TOF MS)

**Figure 1:**
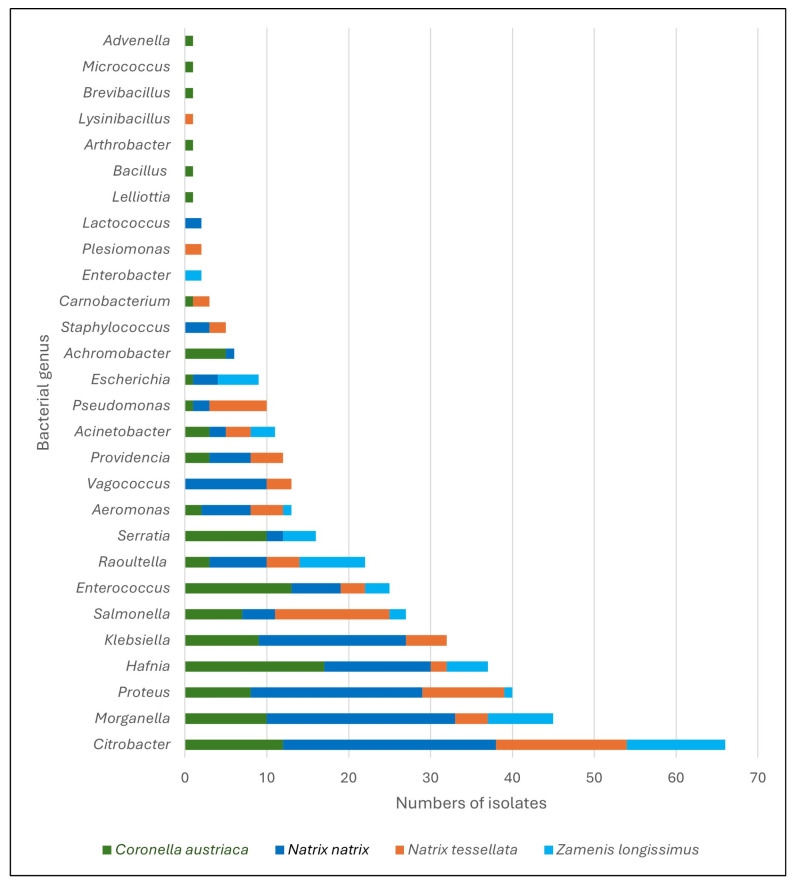
*Genera* of bacteria from cloacal swabs from wild snakes identified using MALDI-TOF MS (*Matrix-Assisted Laser Desorption/Ionization—Time of Flight Mass Spectrometry*).

Among all 432 isolated obtained from wild and kept snakes, the majority (*n* = 417, 96.5%) were identified and classified by MALDI-TOF MS (*Matrix-Assisted Laser Desorption/Ionization—Time of Flight Mass Spectrometry*) into eight taxonomic orders: Enterobacterales (*n* = 319 isolates), Lactobacillales (*n* = 45), Pseudomonadales (*n* = 23), Aeromonadales (*n* = 13), Bacillales (*n* = 7), Burkholderales (*n* = 7), Micrococcales (*n* = 2), and Carynophanales (*n* = 1). Representatives of Enterobacterales, Lactobacillales, and Pseudomonadales were detected in all examined species of free-living snakes (Figure 1). Bacteria of the order Bacillales were found in *N. natrix*, *N. tessellata*, and *C. austriaca*, while Burkholderales were identified exclusively in *N. natrix*. Members of the orders Micrococcales and Carynophanales were isolated only from *C. austriaca*.

A total of 28 different bacterial genera, including both Gram-negative and Gram-positive bacteria, were identified from samples collected from wild snakes. The highest genus-level diversity was observed in *C. austriaca*, from which 22 bacterial genera were isolated. Both examined *Natrix* species exhibited a comparable number of identified genera, with 18 genera isolated from *N. natrix* and 17 from *N. tessellata*. In samples collected from *Z. longissimus*, 12 bacterial genera were detected. The majority of the identified bacteria were Gram-negative. The most frequently isolated bacteria were *Citrobacter* sp. (*n* = 66 isolates), *Morganella* sp. (*n* = 45), *Proteus* sp. (*n* = 40), *Hafnia* sp. (*n* = 37), *Klebsiella* sp. (*n* = 32), *Salmonella* sp. (*n* = 27), *Enterococcus* sp. (*n* = 25), and *Raoultella* sp. (*n* = 22). These genera, along with the less commonly isolated *Aeromonas* sp. (*n* = 13) and *Acinetobacter* sp. (*n* = 11), were detected in all snake species included in the study. Certain bacterial genera were recovered only from *Natrix* species, although this may reflect limitations of culture-based methods. These included Gram-positive *Vagococcus* sp. (*n* = 13) and *Staphylococcus* sp. (*n* = 5), which were isolated from both *Natrix*; *Plesiomonas* sp. (*n* = 2) and *Lysinibacillus* sp. (*n* = 1), found only in *N. tessellata*; and *Lactococcus* sp. (*n* = 2), isolated exclusively from *N. natrix*. Among all genera identified by MALDI-TOF MS, ten were detected exclusively in a single snake species. These included *Lelliottia* sp., *Bacillus* sp., *Arthrobacter* sp., *Brevibacillus* sp., *Micrococcus* sp., *Advenella* sp., all restricted to *C. austriaca*, and *Enterobacter* sp., found only in *Z. longissimus*. From samples collected from captive *E. dione*, only four species were isolated, including *Salmonella enterica* (*n* = 6), *Pseudomonas aeruginosa* (*n* = 2), *Morganella morganii* (*n* = 2) and *Enterococcus faecalis* (*n* = 2). The complete list of identified bacterial species is presented in Table 1.

### 2.3. Detection of Salmonella *spp.* in Snakes

Identification of bacterial isolates using MALDI-TOF MS confirmed the presence of *Salmonella* sp. in all examined species of both wild and captive snakes. *Salmonella* was detected in free-living individuals from four of the eight surveyed field locations, accounting for 50% of all sites included in the study. Notably, in the remaining locations where *Salmonella* spp. was not detected, cloacal samples were collected from only single individuals, which likely reduced the probability of bacterial detection. In total, 32 *Salmonella* spp. isolates were recovered: 14 from *N. tessellata* (from 7 individuals, 37% of all sampled individuals of this species), 6 from *C. austriaca* (3; 13%), 4 from *N. natrix* (3; 12%), and 2 from *Z. longissimus* (2; 22%). An additional 6 isolates were obtained from captive *E. dione* (100% of the individuals of this species included in the study).

#### O Antigen Serotyping of *Salmonella* Isolates

**Figure 2:**
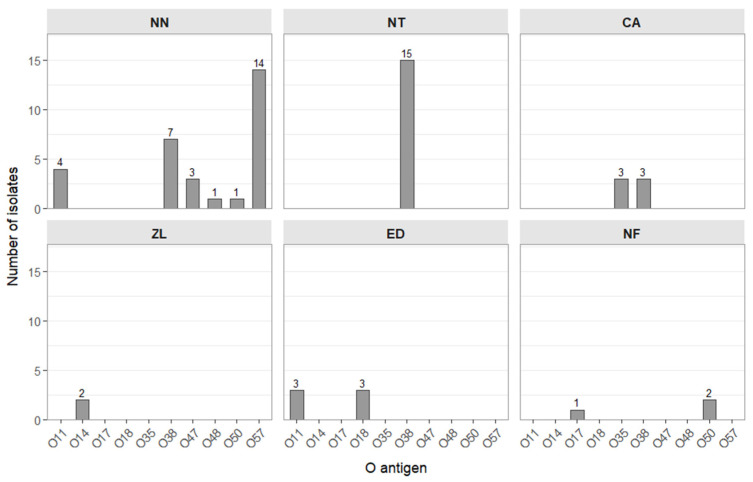
Distribution of O Antigens (O-Ag) in tested *Salmonella* from snakes; NN—*N. natrix;* NT—*N. tessellata;* CA—*C. austriaca;* ZL—*Z. longissimus;* ED—*E. dione;* NF—*N. fasciata.*

All isolates (100%) were assigned to an O antigen variant (*n* = 62). As none reacted with the group-specific sera O:4 (O:B), O:6,7 (C:O); O:9,46 (D:O); O:3,10,15 (E:O), only rarely isolated serovars were present in the studied population. The majority of isolates belonged to antigen groups O:38 (*n* = 26) and O:57 (*n* = 14), which represented 63% of the isolates (Figure 2). In addition, O:11 antigen was detected in seven isolates, while other antigen variants were observed in no more than three isolates each. The occurrence of three O antigen variants (O:11, O:38, and O:50) was confirmed in *Salmonella* isolated from more than one snake species. Among them, O:38 was the most prevalent, being detected in isolates from three wild snake species (*N. tessellata*, *N. natrix*, and *C. austriaca*). Antigens O:11 and O:50 were present in isolates from both wild and captive snakes: O:11 was identified in isolates from *N. natrix* (*n* = 4) and *E. dione* (*n* = 3), whereas O:50 was detected in isolates from *N. fasciata* (*n* = 2) and in one isolate from *N. natrix*. Antigen O:38 was the only O antigen type confirmed in isolates from *N. tessellata*, while O:14 was exclusively detected in isolates from *Z. longissimus*. In the remaining snake species, more than one O antigen variant was identified. In one free-living *N. natrix* individual, the presence of *Salmonella* O:48 was confirmed (isolate NN 26.5)

The coexistence of more than one *Salmonella* serovar within a single snake individual was confirmed. This phenomenon was observed in both tested species of captive snakes, *N. fasciata* and *E. dione*. From individual NF9, three *Salmonella* isolates were obtained, which were classified into two O antigen groups: O:50 and O:17. In the case of *E. dione*, *Salmonella* were isolated from three individuals; in two of them, the coexistence of types O:11 and O:18 was confirmed. No coexistence of more than one *Salmonella* O-antigen group was observed in any of the analyzed wild snake species.

### 2.4. Resistance to Antimicrobials

Susceptibility of *Salmonella* spp. isolates from both free living and captive reptiles (*n* = 32) were assessed against 25 antibiotics and chemotherapeutics. The effectiveness of each antimicrobial was evaluated based on the minimal inhibitory concentration (MIC) values and the results are presented in Table S1. The majority of *Salmonella* spp. were susceptible to all tested antimicrobials (21 isolates; 66%). Resistance to the antimicrobial activity of tigecycline (TIG) was the most frequently observed among all testes *Salmonella* spp. strains. The MIC_TIG_ of all resistant strains was 1 mg/L whereas the MIC_TIG_ of the susceptible strains was ≤0.5 mg/L. Resistant isolates were obtained from both wild snakes—*N. tessellata* (NT 1.8, NT 10.1, NT 18.4) and *C. austriaca* (CA 10.5)—as well as from captive *E. dione* snakes (ED 1.1, ED 1.3, ED 2.3, ED 3.1, ED 3.2). Resistance to amoxicillin/clavulanic acid (AMC), ampicillin (AMP), cephalexin (CF), cefuroxime (CXM), cefixime (CFM), and fosfomycin (FOS) was observed in individual *Salmonella* spp. isolates (Table 2). The MIC_AMC_ for three amoxicillin/clavulanic acid resistant isolates (NT 18.4, CA 10.5, and ZL 1.3) were >32/2 mg/L, whereas MIC_AMC_ of the susceptible was ≤2/2 mg/L. Single cases of resistance to first-, second-, and third generation cephalosporins were also detected with MIC_CF_ > 16 mg/L, MIC_CXM_ > 8 mg/L and MIC_CFM_ of 2 mg/L. One isolate showed resistance to fosfomycin with an MIC_FOS_ of 64 mg/L.

Based on MIC interpretation, five different resistance patterns (RP1 to RP5) were distinguished. List of the RP is presented in Table 2. The most common was RP1, with isolates susceptible to all tested antimicrobials (*n* = 21; 66%). Among isolates showing resistance to at least one antimicrobial, the most frequent was RP2 (*n* = 8; 25%), characterized by resistance to tigecycline. Each of the remaining patterns (RP3, RP4, and RP5) was represented by a single isolate (3%). Analysis of resistance patterns allowed the characterization of isolate NT 18.4 as multidrug-resistant (MDR). This isolate exhibited pattern RP5 and showed resistance to antibiotics from multiple classes, including penicillin, cephalosporins, tigecycline, and fosfomycin.

### 2.5. Resistance to Bactericidal Activity of Human Serum

The survival of *Salmonella* isolates in commercial product of human serum (HS) was evaluated after 3 h of incubation at 37 °C in 50% HS. All isolates were able to grow in the presence of 50% heat-inactivated human serum (IHS), with viable counts exceeding 1.0 × 10^8^ CFU/mL (colony forming units per milliliter). In contrast, when exposed to non-inactivated HS, considerable variation in susceptibility to the bactericidal activity was observed among the tested isolates. The mean number of viable cells was 7.28 × 10^5^ CFU/mL in serum-sensitive strains, 2.35 × 10^6^ CFU/mL in intermediate strains, and 1.47 × 10^8^ CFU/mL in serum-resistant strains at the end of exposure. Most strains (56 isolates) exhibited high serum resistance (R), markedly increasing their viable cell counts during the incubation. In these isolates, the CFU/mL increased from the start of exposure (T_0_) to the end of incubation (T_3_), with survival rates exceeding 1500% relative to the initial inoculum. In contrast, serum-sensitive (S) strains showed a substantial decrease in viability, with survival rates reduced to approximately 12% (*n* = 5). Only one isolate (NT 6.4) represented the intermediate (I) phenotype, maintaining survival rate at around 67% of the starting CFU/mL.

The serum-resistant isolates exhibited continuous proliferation throughout the entire incubation period. The estimated number of bacterial divisions was determined at each time point, showing a gradual increase with prolonged exposure to serum. On average, resistant strains divided approximately 1.68; 3.11 and 4.15 times after 1 h (T_1_), 2 h (T_2_), and 3 h (T_3_) of incubation, respectively. In contrast, sensitive strains showed a consistent decline in viable cell numbers throughout the incubation period. The single intermediate strain exhibited a decrease in CFU/mL after 1 h and 2 h, followed by a slight increase after 3 h of incubation. Detailed mean growth and survival values for all phenotypes are shown in Table 3.

**Figure 3:**
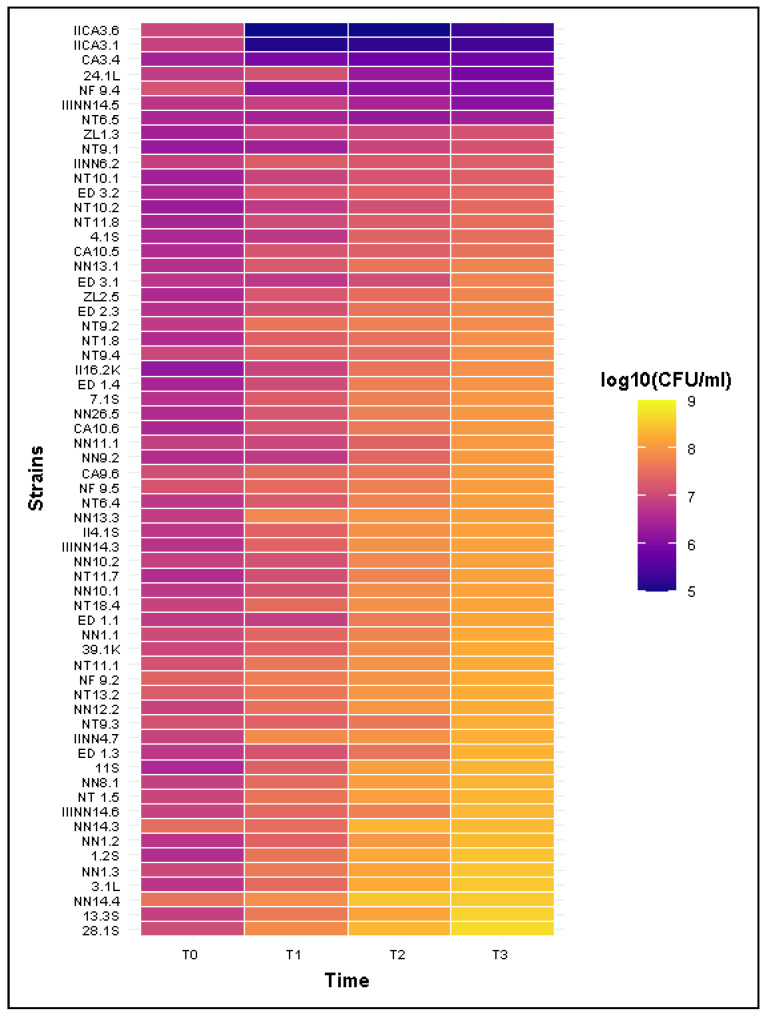
Heatmap of log_10_ CFU/mL values for *Salmonella* isolates incubated in human serum over time.

Among the five sensitive strains, three were isolated from a single individual of *C. austriaca* (CA 3.4. IICA 3.1. IICA 3.6, all belonging to serogroup O:38) two from *N. natrix* (24.1 L, O:50 and IIINN 14.5, O:38), and one from *N. fasciata* (NF 9.5, O:50). Thus, the sensitive phenotype was observed in isolates obtained from both wild and captive snakes. After 3 h of incubation in serum the final viable cell counts of these isolates ranged from 2.00 × 10^5^ to 1.27 × 10^6^ CFU/mL. The single intermediate isolate was isolated from *N. tessellata* (NT 6.5, O:38) and reached 2.35 × 10^6^ CFU/mL after incubation. All remaining isolates displayed a serum-resistant phenotype. These resistant strains originated from all snake species included in the study representing both wild-caught and captive individuals. Among them, 36 isolates exceeded final viable counts of 1.03 × 10^8^ CFU/mL, which corresponded to the mean value observed for the O:48 isolate (NN 26.5). Notably, within the ten isolates showing the highest CFU/mL values, all were derived from wild snakes; nine from *N. natrix* and one from *N. tessellata*. Their final CFU/mL counts exceeded 2.23 × 10^8^, confirming the strong proliferative potential of these strains in the presence of human serum (Figure 3).

## 3. Discussion

Reptiles are frequent carriers of bacteria that are potentially dangerous to humans, especially *Salmonella*. For many years, these animals were not widely recognized as a significant source of pathogens in Europe. In recent years, increased interest has been observed in several European countries, including Spain, Portugal, Italy, and Poland. However, despite the growing awareness of the role of reptiles in the epidemiological chain of zoonoses, most studies still focus on lizards rather than snakes [17]. Notably, most analyses in Europe focus on samples collected from captive individuals [18]. In contrast, the situation is different on other continents, where a much higher proportion of samples is collected from wild animals. Outside Europe, studies are conducted in selected countries of North and South America (mainly the USA and Brazil), as well as in Asia (primarily China and Japan) and Oceania. In all of these regions, samples from wild individuals constitute more than half of all material, with the highest proportions recorded in Australia. Research from Africa is limited, but here as well most studies are based on wild reptiles. Europe is the only continent where samples from captive reptiles clearly predominate over those from wild populations [18].

In our study, the use of the proteomic MALDI TOF MS method to identify bacteria obtained from swabs taken from snakes in Poland allowed us to determine the affiliation of isolates to 28 genera of Gram-negative and Gram-positive bacteria. The most frequently isolated were Gram-negative rods belonging to the genera *Citrobacter, Morganella, Proteus, Hafnia, Klebsiella, Salmonella* and Gram-positive streptococci from the genus *Enterococcus* (Table 1). The presence of *Salmonella* was confirmed in all analyzed snake species, including 19% of the free-living individuals examined. The highest frequency of isolates identified as *Salmonella* was found in kept *E. dione* (*Salmonella* present in 100% of the examined individuals, 3/3) and free-living *N. tessellata* (37%, 7/19) and *Z. longissimus* (22%, 2/9). The lowest frequency was found in *N. natrix* (12%, 3/26) and *C. austriaca* (13%, 3/24). In recent years, only a few studies have addressed the prevalence of *Salmonella* or other bacteria in free-living snakes in Europe [10,19,20]. Up to date, we believe that the results presented in this publication concern the largest group of free-living snakes studied in Europe. In the literature, there are individual studies with very different results. The reason is certainly the different research methodology regarding the type of material collected, the method of sampling, or the technique of identifying the types of bacteria. Comparing our results with those of Schmidt et al. conducted on *N. natrix* (*n* = 12) and *Vipera berus* (*n* = 23) living freely in Germany, we find no correlation [20]. *Salmonella* was not detected in *N. natrix.* while in *V. berus* it constituted 34.8% of the isolated microbiota. Considering the lower number of individuals studied and the use of different methods, it is difficult to compare these results with those obtained by our team. It should be highlighted, that free-living snakes are a particularly difficult group to study. They are rare, secretive, migratory animals, often inhabiting areas difficult to access by humans. Therefore, the research model has numerous limitations, and repeatability of methods and the number of individuals is difficult to achieve. Each field trip is time-consuming and results in finding a different number of individuals. Due to the speed and movement of these animals, not every snake encountered is successfully captured. The microbiological studies we presented were performed in conjunction with expert herpetological examinations and were dependent on them. There is no gold standard for reptile microbiota testing. Test material also varies, for example, cloacal swabs or fecal samples. Some studies focus on culturing methods and isolating as many bacterial species as possible, others only examine the number of Gram-negative species, and still others solely isolate *Salmonella* according to ISO standards. In each case, different microbiological media will be used, selective for specific microbial groups, so the results of such tests cannot be compared. Also, completely different results will be obtained when molecular biology methods are used instead of culturing methods. For this reason, research on the reptile microbiota should be continued and expanded, as each result obtained significantly enriches knowledge [18,21].

Among the bacteria colonizing reptiles, *Salmonella* rods are of major clinical relevance, and the subject of research in most scientific publications, as they are responsible for RAS in humans. These infections mainly affect immunocompromised patients and are frequently associated with extraintestinal diseases which require hospitalization, such as sepsis or meningitis [22,23]. In 2023, Bruning et al. described a case of reptile-associated urinary tract infection in an 18-year-old woman caused by *Salmonella* Oranienburg, while in 2021, Otake et al. reported a case of testicular necrosis due to *Salmonella* Saintpaul following contact with a snake [24,25]. Despite limited RAS surveillance in Europe, such cases are nevertheless diagnosed. For instance, a case report from Austria documented an infection with *Salmonella enterica* subsp. *enterica* ser. Monschaui (O:35) in a three-week-old infant [26]. Unlike Europe, North American countries, such as the USA and Canada, conduct RAS monitoring. Important outbreaks are reported by relevant authorities, including the Centers for Disease Control and Prevention (CDC). Although less frequent, other bacteria have also been implicated in snake-associated human infections. Among the most frequently documented non-RAS cases are wound infections resulting from snake bites, caused by *Morganella*, *Enterobacter*, *Klebsiella*, *Aeromonas hydrophila*, and various other Gram-positive and Gram-negative bacteria [27,28]. These examples highlight the underappreciated zoonotic potential of snake-associated microbiota and underscore the importance of a One Health perspective in recognizing and managing such infections [21,29,30].

*Salmonella* are highly diverse with respect to surface antigenic variants, and more than 2600 serovars have currently been distinguished on this basis. Serological variability directly contributes to differences in the pathogenicity of these bacteria [31]. In our study, *Salmonella* isolates were assigned to the O-antigen serotypes O:11, O:14, O:17, O:18, O:30, O:35, O:38, O:47, O:48, O:50 and O:57. The most frequently identified were O:38, present in all isolates from *N. tessellata* and in three isolates from *C. austriaca*, and O:57, which was exclusively confirmed in *N. natrix*. All of these variants are considered reptile-associated. At the same time, these O groups are rarely reported from patients in Poland and Europe, where the majority of salmonellosis are caused by serovars O:9,46 (*S*. Enteritidis), O:4 (*S.* Typhimurium, *S.* Derby), and O:6,7 (*S.* Infantis, *S.* Virchow, *S.* Newport [4,32,33]. Rare serovars are isolated in approximately 1–2% of diagnosed salmonellosis cases in Poland, depending on the reporting year [32]. This low frequency may be partly explained by distinct biochemical features, particularly the ability of *Salmonella enterica* subsp. *diarizonae* to ferment lactose, a feature characteristic of reptile-derived isolates, which we also confirmed in our previous [34]. Clinical isolates belonging to these rare O groups remain poorly studied, and in our view, there is an urgent need for their further characterization. Detailed molecular descriptions of these O antigens are limited; among the few characterized variants is O:48. In the present study, we confirmed the presence of *Salmonella* O:48 in one isolate from *N. natrix*. Flagellar antigen serotyping suggested that this strain may belong to serovar 48:k:z_57_ or a closely related variant (unpublished data). *Salmonella* 48:k:z_57_ has previously been identified in human cases in Poland [31]. Structural analyses of the lipopolysaccharide (LPS) of *Salmonella* O:48, conducted by Gamian et al., revealed its unique composition, characterized by the presence of sialic acid residues within its structure [35]. Pawlak et al. demonstrated that elongation of the O:48 antigen following serial passage in human serum correlated with increased resistance to its bactericidal activity [36]. Such a structural feature may enhance the clinical importance of these variants, as it can contribute to post-infectious complications through molecular mimicry [37]. Data on the occurrence of *Salmonella* in the snake species examined in our study are mainly limited to *N. natrix*, and there is a lack of information on potential RAS cases associated with these species [11,19,33]. However, useful supplementary evidence can be found in EnteroBase [38]. Individual records confirm the presence of *Salmonella* Newport in kept *Zamenis situla* in Poland. No records refer to individuals of wild species. Furthermore, EnteroBase data confirm that several *Salmonella* serogroups detected in our study are also frequently isolated from human clinical cases. For instance, numerous human isolates belonging to *Salmonella* O:38 have been reported, including variants such as 38:r:z and 38:k:z_35_. Human isolates representing serogroup O:50, such as 50:k:z and 50:r:z, and those belonging to serogroup O:57, including 57:c:z and the monophasic variant 57:z_4,_z_32_:-, have also been documented. The data also confirm the isolation of *Salmonella* O:48, which we detected in free-living *N. natrix*. Reported human isolates include serovars such as 48:i:z, 48:d:z_6_, 48:l,v:1,5 and 48:k:z_53_, with the latter documented also in Poland [31,38]. Overall, the serogroups identified in our study align with those periodically isolated from human cases, indicating that the strains carried by these snakes may contribute to the broader pool of *Salmonella* circulating in the environment and occasionally affecting humans. In addition to identifying serologic groups that have previously been reported in Europe, it is equally important from an epidemiological perspective to characterize their antimicrobial-resistance profiles. Such resistance is one of the key determinants of pathogenic potential of individual strains and influences their role in the transmission chain of zoonotic infections. This aspect becomes particularly important given that antimicrobial resistance among Enterobacterales, including *Salmonella*, represents a major global public health challenge.

The problem has been recognized by the World Health Organization (WHO), which considers the increasing prevalence of resistance to be a serious threat. In the 2024 WHO bacterial priority pathogens list (BPPL), both typhoidal and non-typhoidal *Salmonella* resistant to fluoroquinolones were classified as pathogens of the high-priority group [39]. Wet markets have been identified as one of the sources of resistant strains. Alarmingly, data from South Asia indicate a high proportion of MDR *Salmonella* isolates recovered from wet market samples in Bangladesh [40]. In our study, the majority of isolates (66%) were susceptible to all tested antimicrobials. Among all isolates, five different resistance patterns (RP1–RP5) were identified. One isolate (NT 18.4) from *N. tessellata* exhibited the RP5 profile and was classified as MDR, showing resistance to β-lactams (penicillins: amoxicillin–clavulanic acid and penicillin; first- and second-generation cephalosporins: cephalexin and cefuroxime), as well as to tigecycline and fosfomycin. We also observed that 28% of isolates were resistant to tigecycline (RP2 and RP5 patterns). Tigecycline-resistant strains were isolated from both wild (*N. tessellata*. *C. austriaca*) and captive snakes (*E. dione*). This finding confirms our previous results, where tigecycline resistance was detected in five isolates from *N. natrix* and one from *N. fasciata* (20% of tested isolates), further highlighting the occurrence of this resistance phenotype in *Salmonella* from both wild and captive snake populations [34]. Bertelloni et al. reported a high percentage (93.1%) of tigecycline non-sensitive *Salmonella* strains isolated from reptiles [41]. Efflux activity and the presence of genetic determinants have been identified as the main mechanisms underlying reduced tigecycline susceptibility in *Salmonella*. In particular, the AcrAB–TolC efflux pump system and the plasmid-mediated tigecycline resistance gene *tet(X4)* have been associated with this phenotype [42,43]. In a recent study, El-Aziz et al. correlated elevated MIC values for both tigecycline and ciprofloxacin with increased efflux pump activity, further highlighting the central role of efflux in multidrug resistance [44]. In our isolates, the *tolC* gene was detected in all strains; for half of them, these results have already been reported in our previous publication [34]. The presence of *tolC* may contribute to the observed resistance profiles within snake-associated *Salmonella* populations, as this gene encodes an essential component of multidrug efflux systems. Confirmation of *tolC* expression and characterization of the activity of the corresponding efflux pump under different environmental conditions constitute one of our forthcoming research objectives.

Various environments play an important role as reservoirs of antibiotic-resistant bacteria. Aquatic habitats are frequently highlighted as important reservoirs, and the role of wildlife in the transmission of antimicrobial-resistance genes has also been emphasized [45,46]. Although reptiles are rarely discussed in this context, the presence of resistant strains in wildlife is documented [47]. Snakes as animals that interact with aquatic environments and feed on small vertebrates and invertebrates, reptiles may represent a potential link in the trophic transmission of antimicrobial-resistance determinants [48]. As indicated, the presence of antibiotic-resistant, reptile-associated *Salmonella* strains has already been confirmed in the literature [49,50]. This process may be further enhanced by the fact that many resistance genes in *Salmonella* are plasmid-encoded, facilitating their horizontal transfer [51].

Most of the tested strains were resistant to bactericidal action of 50% HS. Studies by Dudek et al. and Nishio et al. confirmed that the serum-resistant phenotype of *Salmonella* is associated with the composition of outer membrane proteins (OMPs), including PagC and OmpA [52,53]. In addition to OMP composition, numerous studies have demonstrated that lipopolysaccharide (LPS) structure strongly influences complement resistance, and this effect appears to be common to both non-typhoidal and typhoidal *Salmonella* [54,55]. In addition, Pawlak et al. showed, using *Salmonella* O:48 as an example, that resistance to human serum correlates with LPS chain elongation [36]. The serum resistance phenotype may also be associated with the presence of specific plasmids [56]. Given the complex and multifactorial nature of human serum resistance, further studies on reptile-associated *Salmonella* are warranted, particularly considering their potential zoonotic significance.

## 4. Materials and Methods

### 4.1. Collection of the Cloacal Swabs from Snakes

**Figure 4:**
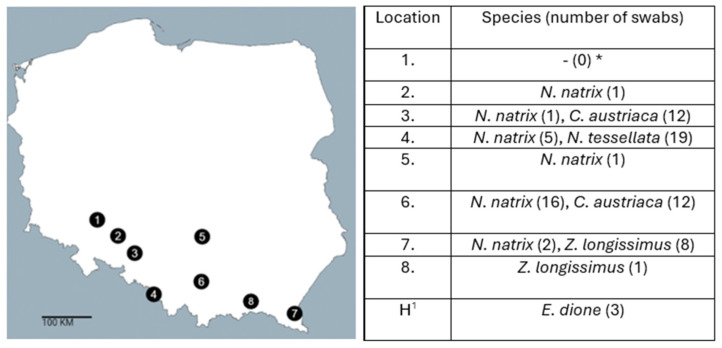
Locations of sampling and numbers of samples collected from each species. Locations near to cities: Wroclaw—location 1, Gogolin—3, Cieszyn—4, Włoszczowa—5, Kraków—6, Teleśnica Oszwarowa—7, Dukla—8 and Babi Loch Lake—2; (*) No snakes were found at location 1 during sampling; (^1^) H—Swabs collected from captive snakes (private breeders) in Kraków (location no. 6).

Snakes were captured in eight different regions in Poland as shown on Figure 4. The fieldwork was designed as an exploratory study. Bacteriological swabbing was added as an additional component, and the sampling depended on collaboration with field experts. Moreover, higher snake densities in southern Poland further influenced the spatial distribution of sampling. Field surveys were conducted from 2021 to 2024 during the active season of these animals, which is mainly April to October. However, bacteriological swabs were successfully collected only in 2021, 2022, and 2023, with 24, 25, and 19 samples obtained in those years, respectively. Because snakes are typically secretive and occur at low densities, sampling is challenging. For this reason, all encountered individuals were swabbed except for newly hatched ones. No additional exclusion criteria were applied. In total, 78 cloacal swabs from free-living snakes, including 26 *N. natrix*, 24 *C. austriaca,* 19 *N. tessellata* and 9 *Z. longissimus* were collected. Each individual was sampled once, except for the *C. austriaca* individual designated CA 3 which was captured and swabbed twice during two independent field surveys. Additionally, swabs from 3 breeding *E. dione* were collected. Prior to conducting the study, the necessary permits were obtained consents from the Regional Directorates for Environmental Protection in Wrocław, Kraków, Katowice and Opole (decisions no. WPN.6401.61.2021.AP, no. WPN.6401.140.2021.AR, no. WPN.6401.270.2019.ZB, no. OP.6401.77.2021.KW), as well as from the General Directorate for Environmental Protection in Warsaw (decision no. DZP-WG.6401.91.2020.TŁ, DZP-WG.6401.80.2023.TŁ.2). Swabs were collected in a non-invasive manner, during other measurement-related work carried out in the field. Due to the non-invasive character of the sampling, and in accordance with the Act of 15 January 2015 on the Protection of Animals Used for Scientific or Educational Purpose, approval from local ethics committee was not required [57]. To prevent pathogen transmission, particularly of snake fungal disease (SFD), strict biosafety measures were followed during sample collection. Cloacal swabs were collected by inserting the swab into the cloaca and rotating it 360° three times to collect microbiological material. Swabs were then placed in Amies transport medium, stored at room temperature and transported to the Department of Microbiology, University of Wroclaw for bacteriological examination.

### 4.2. Bacteriological Examination

Swabs collected from snakes were first enriched in Buffered Peptone Water (Biomaxima, Lublin, Poland) and incubated at 37 °C for 18 h with shaking at 160 rpm to promote bacterial growth. Following incubation, bacteria were streaked onto non-selective (Nutrient Agar), and selective (MacConkey) solid media (Biomaxima, Lublin, Poland). Individual bacterial colonies were selected based on distinct morphological characteristics and lactose fermentation profiles on MacConkey Agar, ensuring the isolation of diverse bacterial strains. The isolated colonies were then suspended in LB medium (Biomaxima, Lublin, Poland) supplemented with 25% (*v*/*v*) glycerol and stored at −70 °C until further identification. 

### 4.3. MALDI-TOF MS Identification

The proteomic method MALDI TOF MS was used to identification of the collected bacterial strains (*n* = 432). Ribosomal proteins of each tested strain were extracted with ethanol and formic acid for protein detection and identification. Two to five colonies were harvested from Nutrient Agar (Biomaxima, Lublin, Poland) plates after 24 h of incubation at 37 °C and suspended in 300 µL of distilled water. Then, 900 µL of pure ethanol was added and the samples were homogenized using vortex mixer for 60 s followed by centrifugation at 13,000× *g* for 2 min. After removing the supernatant, the bacterial pellet was air-dried at room temperature to ensure complete evaporation of residual ethanol. The dried pellet was then resuspended in 25 µL of a 70% formic acid solution and 25 µL of acetonitrile (Sigma-Aldrich, St. Louis, MO, USA), mixed thoroughly, and centrifuged (2 min, 13,000× *g*) to extract ribosomal proteins. For protein identification, 1 µL of the supernatant from each sample was transferred onto a steel MSP 96-target polished plate and air-dried at room temperature. Then, 1 µL of HCCA matrix (α-Cyano-4-hydroxycinnamic acid, (Sigma-Aldrich, St. Louis, MO, USA) was added to an each sample spot and dried again. The plates were analyzed using MALDI Biotyper Sirius mass spectrometer (Brucker, Bremen, Germany) and the mass spectra of extracted proteins were obtained with flexControl Version 3.4 software. Mass spectral identification was performed using the MBT Compass 4.1 and MALDI Biotyper Compass Explorer 4.1 software were used. Identification scores were assigned by comparing the mass spectral profiles of the analyzed samples with reference spectra from a curated database. The MBT Compass Library Revision K (2022) database (Bruker Daltonics GmbH & Co. KG, Bremen, Germany) containing 4274 species were used. Identification results were classified according to the manufacturer’s guidelines as follows: 

Score: 2.300–3.000: Highly probable species identification

Score: 2.000–2.999: Secure genus identification, probable species identification

Score: 1.700–1.999: Probable genus identification

Score: 0.000–1.699: No identification

Although bacterial identification was performed for all isolates, only *Salmonella* spp. were selected for further analysis due to their established role in RAS and potential for zoonotic transmission. All isolates identified in this work as *Salmonella* (*n* = 32) were included in the subsequent analyses, together with 30 isolates previously obtained from NN and NF, resulting in a total of *n* = 62 [34]. Antibiotic resistance testing was conducted exclusively for the isolates described in the present study, as data for the previously published isolates have been reported [34].

### 4.4. Determination of O Antigen in Salmonella *spp.* Isolates

All isolates previously identified as *Salmonella* sp. by MALDI-TOF MS (*n* = 62) were subjected to O antigen serotyping with slide agglutination method according to ISO/TR 6579-3: *Microbiology of the food chain—Horizontal method for the detection, enumeration and serotyping of Salmonella—Part 3: Guidelines for serotyping of Salmonella* spp. and the Kaufmann–White–Le Minor scheme [58,59]. Fresh colonies grown for 18 ± 2 h at 37 °C on non-selective Nutrient Agar were used for serological testing. Rough strains were identified by suspending colonies in 0.9% NaCl and autoagglutination-positive isolates were discarded. The remaining isolates were confirmed with polyvalent HM serum, specific for all *Salmonella* serovars. Polyvalent and monovalent antisera from different manufacturers were used, including OMC, OME, OMF, OMG (BioRad, Marnes-la-Coquette, France); OMD, O:4 (formerly B:O), O:6,7 (C:O); O:9,46 (D:O); O:3,10,15 (E:O); O:11, O:14, O:21 (Immunolab, Gdansk, Poland); O:6, O:14, O:16, O:17, O:18, O:28, O:35, O:38, O:47, O:50, O:51, O:52, O:53, O:56, O:57, O:58, O:59, O:61 (Sifin, Berlin, Germany) and O:48 (SSI Diagnostica, Hillerød, Denmark). For each agglutination reaction, a small *Salmonella* spp. inoculum consisting of 2–3 colonies was suspended in a drop of the respective serum placed on a glass slide and spread until a homogeneous suspension without visible clumps was obtained, as clumping could lead to false-positive results. The suspension was then gently tilted back and forth on the slide for approximately 5–60 s, with the exact time adjusted according to the manufacturer’s instructions for the specific serum. Agglutination results were read under indirect light against a dark background. A positive reaction was defined as the visible formation of antigen–antibody aggregates within the drop, whereas a negative reaction was indicated by a homogenous, milky appearance of the suspension.

### 4.5. Antimicrobial Susceptibility Testing (AST) 

The BD Phoenix^TM^ M50 (Becton Dickinson, Sparks, MD, USA) automated system was used for antimicrobial susceptibility testing (AST). All isolates classified as *Salmonella* sp. based on the MALDI-TOF MS and slide agglutination procedures were subjected to AST (*n* = 32). Antimicrobial susceptibility results for the remaining 30 isolates from *N. natrix* and *N. fasciata* were reported in our previous work [34]. The NMIC-502 panel was selected to determine the minimum inhibitory concentration (MIC) of the following 26 antimicrobial agents (concentration ranges in mg/L): amoxicillin/clavulanic acid (2–32); ampicillin (2–8); piperacillin (4–64); piperacillin/tazobactam (4–64); temocillin (4–32); cephalexin (4–16); cefepime (1–16); cefixime (0.5–2); ceftazidime (0.5–8); ceftazidime/avibactam (0.25/4–8/4); ceftriaxone (0.5–4); cefuroxime (2–8); ertapenem (0.25–1); imipenem (0.25–8); meropenem (0.125–8); ciprofloxacin (0.06–1); levofloxacin (0.5–2); amikacin (4–16); gentamicin (1–4); tobramycin (1–4); tigecycline (0.5–2); aztreonam (1–16); fosfomycin (16–128); colistin (0.5–2); nitrofurantoin (16–64); trimethoprim-sulfamethoxazole (1–4). According to the manufacturer’s specifications, the NMIC-502 panel is designed for the determination of susceptibility profiles across a broad range of antibiotics relevant to Gram-negative bacteria, including both fermentative and non-fermentative species. The BD Phoenix™ M50 system utilizes a broth microdilution assay for AST. Each panel includes a wide range of two-fold serial dilutions of multiple antimicrobial agents, allowing for precise MIC determination. Bacterial growth was continuously monitored by detecting redox indicator changes and variations in turbidity, ensuring reliable assessment of antimicrobial susceptibility. A 24 h culture of each *Salmonella* strain on a Nutrient Agar was used for the testing. The inoculum was prepared by suspending several colonies in a sterile saline solution (ID test tube) to obtain a 0.5 McFarland standard, corresponding to approximately 1.5 × 10^8^ CFU/mL. The turbidity of the suspension was measured using a densitometer. A drop of AST Indicator Solution and 25 µL of the bacterial suspension were transferred to an AST broth test tube. The inoculated AST broth was then dispensed into the selected NMIC-502 panel, which was subsequently placed into the instrument and incubated at 35–37 °C for 16–18 h. The obtained MIC values for each tested antibiotic were interpreted according to the current EUCAST guidelines (European Committee on Antimicrobial Susceptibility Testing) [60]. Strains were classified as “S” (Susceptible, standard dosing regimen), “I” (Susceptible, increased exposure), or “R” (Resistant).

### 4.6. Sensitivity to Bactericidal Activity of HS

The ability of the tested *Salmonella* strains to resist the bactericidal activity of HS was assessed by quantifying bacterial survival following exposure to 50% HS. A commercial human serum preparation was used (Sigma-Aldrich, Product No. P2918, St. Louis, MO, USA). To ensure consistency of results and preserve maximum serum activity, the serum was aliquoted into 1 mL portions and stored at −70 °C. Each aliquot was defrosted only once immediately before use. HS activity was verified using the reference strain *Salmonella enterica* subsp. *enterica* serovar Typhimurium ATCC 14028, whose sensitivity to the bactericidal effect of human serum had been confirmed in our previous experiments (unpublished data).

A total of 62 *Salmonella* isolates were tested in the HS assay. Bacterial cultures were prepared as previously described by Pawlak et al., and the survival was quantified by the Miles and Misra drop plate technique [34,61]. Briefly, each tested *Salmonella* spp. strain was subcultured on Nutrient Agar for 24 h at 37 °C before testing. Single colonies were then inoculated into 5 mL of LB broth and incubated at 37 °C for 18 h with shaking at 160 rpm. Then, 100 µL of the overnight bacterial culture was transferred into 5 mL of fresh LB broth and incubated again (37 °C, 160 rpm) until reaching 0.5 McFarland standard, corresponding to approximately 1.5 × 10^8^ CFU/mL. The fresh bacterial culture was then centrifuged at 4000 rpm for 20 min at 4 °C. The bacterial pellet was resuspended in 5 mL of sterile physiological saline. From this suspension, 1 mL was further diluted in 3 mL of physiological saline. 

For the bactericidal activity testing, 125 µL of the bacterial inoculum was mixed with an equal volume of human serum, achieving a final serum concentration of 50% (*v*/*v*). The mixture was incubated at 37 °C for 3 h. To monitor bacterial survival over incubation time, serial dilutions were plated at 0 min (T_0_), 60 min (T_1_), 120 min (T_2_), and 180 min (T_3_). At each time point, 10-fold serial dilutions (from 10^−1^ to 10^−6^) were prepared in sterile physiological saline. After overnight incubation at 37 °C, the CFU/mL were enumerated to determine bacterial viability. Each strain was tested in three independent biological replicates, with each biological replicate analyzed in triplicate technical spots. Bacterial survival at T_3_ (180 min) was determined by comparing CFU/mL counts at T_3_ to the initial CFU/mL counts at T_0_ (0 min), providing a measure of serum resistance. A control experiment was conducted for each strain using heat-inactivated serum (56 °C for 30 min), prepared under the same conditions.

## 5. Conclusions

This study demonstrated that wild snakes in Poland constitute a reservoir of *Salmonella*. These are the first confirmed findings of *Salmonella* occurrence in *C. austriaca*, *Z. longissimus*, and *N. tessellata* living in the wild. The isolates represented rare serovars belonging to distant serological groups and showed the ability to multiply in human serum, indicating their potential to survive and proliferate in the presence of immune system components.

## Figures and Tables

**Table 1:** Bacterial microbiota from cloacal swabs of tested snakes in Poland identified using MALDI-TOF MS.

Bacterial Species ^1^	Free-Living Snakes (*n* = 405)				Kept Snakes (*n* = 12)
	NN	NT	CA	ZL	ED
*Achromobacter xylooxidans*	1	-	5	-	-
*Acinetobacter calcoaceticus*	2	1	3	3	-
*A. courvalinii*	-	-	1	-	-
*A. lactuace*	-	2	-	-	-
*Advenella inecata*	-	-	1	-	-
*Aeromonas eucrenophila*	1	-	-	-	-
*A. hydrophila*	4	3	-	-	-
*A. jandaei*	-	-	1	-	-
*A. media*	-	-	-	1	-
*A. veronii*	1	1	1	-	-
*Arthrobacter* sp.	-	-	1	-	-
*Bacillus* sp.	-	-	1	-	-
*Brevibacillus* sp.	-	-	1	-	-
*Carnobacterium* sp.	-	1	-	-	-
*C. maltaromaticum*	-	1	1	-	-
*Citrobacter* sp.	-	1	-	2	-
*C. braakii*	10	5	9	4	-
*C. freundii*	15	10	3	5	-
*C. gillenii*	1	-	-	1	-
*Escherichia coli*	3	-	1	5	-
*Enterobacter cloacae*	-	-	-	1	-
*E. ludwugu*	-	-	-	1	-
*Enterococcus* sp.	1	1	1	-	-
*E. faecalis*	5	2	12	3	2
*Hafnia alvei*	13	2	17	5	-
*Klebsiella aerogenes*	1	-	-	-	-
*K. oxytoca*	17	5	9	-	-
*Lactococcus garviae*	2	-	-	-	-
*Lelliottia amnigena*	-	-	1	-	-
*Lysinibacillus fusiformis*	-	1	-	-	-
*Micrococcus luteus*	-	-	1	-	-
*Morganella morgannii*	23	4	10	8	2
*Plesiomonas shigelloides*	-	2	-	-	-
*Proteus* sp.	-	1	1	-	-
*P. vulgaris*	21	6	8	1	-
*P. hauserii*	-	3	-	-	-
*Providencia* sp.	3	-	1	-	-
*P. rettgerii*	1	4	2	-	-
*P. vermicola*	1	-	-	-	-
*Pseudomonas aeruginosa*	1	7	1	-	2
*P. mendocina*	1	-	-	-	-
*Raoultella ornithinolytica*	6	2	3	3	-
*R. planticola*	1	2	-	4	-
*R. terrigena*	-	-	-	1	-
*Salmonella enterica* ^2^	3	15	6	2	6
*Serratia fonticola*	1	-	-	-	-
*S. liquefaciens*	1	-	2	4	-
*S. marcescens*	-	-	7	-	-
*Staphylococcus* sp.	1	-	-	-	-
*S. sciuri*	1	2	-	-	-
*S. warneri*	1	-	-	-	-
*Vagococcus fluvialis*	10	3	-	-	-

^1^ Isolates with scores between 2.000 and 3.000 were considered reliably identified to the species level and are listed accordingly. For scores in the range of 1.700 to 1.999, only genus-level identification was accepted, and species names are not provided in the table; ^2^ All *Salmonella* isolates were identified with scores 2.000 or higher; however, due to limitations of the MALDI-TOF MS, all results are reported at the genus level (*Salmonella* sp.). Our further analyses confirmed that all *Salmonella* isolated belong to *Salmonella enterica* (unpublished data); NN—*N. natrix;* NT—*N. tessellata;* CA—*Coronella austriaca;* ZL—*Z. longissimus;* ED—*E. dione.*

**Table 2:** Resistance patterns (RP) of tested *Salmonella* isolates.

*n*	%	Susceptible	Resistance	Resistant Pattern
21	66%	AMP-AMC-PIP-TZP-TMO-FEP-CFM-CAZ-CZA-CRO-CXM-CF-ERT-IMP-MER-AZE-CIP-LEV-AK-GEN-TOB-TIG-C-SXT-FOS-NF	-	RP1
8	25%	AMP-AMC-PIP-TZP-TMO-FEP-CFM-CAZ-CZA-CRO-CXM-CF-ERT-IMP-MER-AZE-CIP-LEV-AK-GEN-TOB-C-SXT-FOS-NF	TIG	RP2
1	3%	PIP-TZP-TMO-FEP-CFM-CAZ-CZA-CRO-CXM-CF-ERT-IMP-MER-AZE-CIP-LEV-AK-GEN-TOB-TIG-C-SXT-FOS-NF	AMC-AMP	RP3
1	3%	AMP -PIP-TZP-TMO-FEP-CAZ-CZA-CRO-CXM-CF-ERT-IMP-MER-AZE-CIP-LEV-AK-GEN-TOB-TIG-C-SXT-FOS-NF	AMC-CFM	RP4
1	3%	PIP-TZP-TMO-FEP-CFM-CAZ-CZA-CRO-ERT-IMP-MER-AZE-CIP-LEV-AK-GEN-TOB-C-SXT-NF	AMC-AMP CXM-CF-TIG-FOS	RP5

AMP—ampicillin; AMC—amoxicillin/clavulanic acid; PIP—piperacillin, TZP piperacillin/tazobactam; AZE—aztreonam; FEP—cefepime; CFM—cefixime; CAZ—ceftazidime; CZA—ceftazidime/avibactam; CRO—ceftriaxone; CXM—cefuroxime; CF—cephalexin; IMP—imipenem; ERT—ertapenem; MEM—meropenem; TMO—temocillin; AK—amikacin; GEN—gentamicin; TOB—tobramycin; CIP—ciprofloxacin; LEV—levofloxacin; C—colistin; TGC—tigecycline; SXT—trimetoprim/sulfamethoxazole; FOS—fosfomycin; NF—nitrofurantoin.

**Table 3:** Growth and survival parameters of *Salmonella* phenotypes in human serum after different incubation time.

Phenotype	Parameter	T_1_	T_2_	T_3_
Sensitive(*n* = 5)	Mean CFU/mL ± SD	3.94 × 10^6^ ± 5.48 × 10^6^	1.18 × 10^6^ ± 1.13 × 10^6^	7.28 × 10^5^ ± 4.33 × 10^5^
	Median CFU/mL	1.14 × 10^6^	9.56 × 10^5^	7.86 × 10^5^
	Mean Survival Rate (%)	63.45 ± 84.11%	19.81 ± 20.56%	12.31 ± 10.09%
	Mean number of Estimated divisions	−3.42 ± 2.27	−3.58 ± 1.51	−3.42 ± 2.27
Intermediate (*n* = 1)	CFU/mL ± SD	2.70 × 10^6^	1.82 × 10^6^	2.35 × 10^6^
	Survival Rate (%)	77.14%	51.90%	67.14%
	Number of Estimated divisions	−0.37	−0.95	−0.57
Resistant (*n* = 56)	Mean CFU/mL ± SD	2.55 × 10^7^ ± 1.80 × 10^7^	7.37 × 10^7^ ± 5.97 × 10^7^	1.47 × 10^8^ ± 1.02 × 10^8^
	Median CFU/mL	2.19 × 10^7^	5.56 × 10^7^	1.33 × 10^8^
	Mean Survival Rate (%)	>100%	>1000%	>1500%
	Number of Estimated divisions	1.68 ± 0.77	3.11 ± 0.97	4.15 ± 0.97

CFU/mL—colony forming units per milliliter; SD—standard deviation.

## Data Availability

Data on antibiotic susceptibility (MIC values) are provided in the Supplementary Materials. MALDI-TOF MS identification results and BD PhoenixTM M50 antimicrobial susceptibility reports are available from the corresponding author upon reasonable request.

## Supplementary Materials

The following supporting information can be downloaded at https://www.mdpi.com/article/10.3390/ijms262412018/s1.

## Abbreviations

The following abbreviations are used in this manuscript:

NN	*Natrix natrix*
NT	*Natrix tessellata*
CA	*Coronella austriaca*
ZL	*Zamenis longissimus*
ED	*Elaphe dione*
NF	*Nerodia fasciata*
HS	Human serum
CDC	Centers for Disease Control and Prevention
NTS	Non-typhoidal salmonellosis
pSV	*Salmonella* virulence plasmid
