# Supplementary material for: From Bacterial Diversity to Zoonotic Risk: Characterization of Snake-Associated Salmonella Isolated in Poland with a Focus on Rare O-Ag of LPS, Antimicrobial Resistance and Survival in Human Serum
Source: Int J Mol Sci. 2025 Dec 13;26(24):12018. doi: 10.3390/ijms262412018 (PMC12733340; doi:10.3390/ijms262412018)
Supplement: Supplementary file 1 [file ijms-26-12018-s001.zip › ijms-3997216-supplementary.pdf]

Table S1. Minimal inhibitory concentrations of the tested antimicrobials

Strain	MIC of antimicrobials [mg/L]*																								
	AMP	AMC	PIP	TZP	AZE	FEP	CFM	CAZ	CZA	CRO	CXM	CF	IMP	ERT	MEM	TMO	AK	GE	TOB	CIP	LEV	C	TGC	SXT	FOS	NF
NN 10.1	≤2	≤2/2	≤4	≤4/4	≤1	≤1	≤0,5	≤0,5	≤0,25/4	≤0,5	≤2	≤4	0,5	≤0,25	≤0,125	8	≤4	≤1	≤1	≤0,0625	≤0,5	≤0,5	≤0,5	≤1/19	≤16	32
NN 10.2	≤2	≤2/2	≤4	≤4/4	≤1	≤1	≤0,5	≤0,5	≤0,25/4	≤0,5	≤2	≤4	0,5	≤0,25	≤0,125	8	≤4	≤1	≤1	≤0,0625	≤0,5	≤0,5	≤0,5	≤1/19	≤16	32
NN 11.7	≤2	≤2/2	≤4	≤4/4	≤1	≤1	≤0,5	≤0,5	≤0,25/4	≤0,5	≤2	≤4	0,5	≤0,25	≤0,125	8	≤4	≤1	≤1	≤0,0625	≤0,5	≤0,5	≤0,5	≤1/19	≤16	32
NN 26.5	≤2	≤2/2	≤4	≤4/4	≤1	≤1	≤0,5	≤0,5	≤0,25/4	≤0,5	≤2	≤4	0,5	≤0,25	≤0,125	16	≤4	≤1	≤1	≤0,0625	≤0,5	≤0,5	≤0,5	≤1/19	≤16	≤16
NT 1.5	≤2	≤2/2	≤4	≤4/4	≤1	≤1	≤0,5	≤0,5	≤0,25/4	≤0,5	≤2	≤4	0,5	≤0,25	≤0,125	≤4	≤4	2	2	≤0,0625	≤0,5	≤0,5	≤0,5	≤1/19	≤16	≤16
NT 1.8	≤2	≤2/2	≤4	≤4/4	≤1	≤1	≤0,5	≤0,5	≤0,25/4	≤0,5	≤2	≤4	0,5	≤0,25	≤0,125	≤4	≤4	2	2	≤0,0625	≤0,5	≤0,5	1	≤1/19	≤16	≤16
NT 6.4	≤2	≤2/2	≤4	≤4/4	≤1	≤1	≤0,5	≤0,5	≤0,25/4	≤0,5	≤2	≤4	0,5	≤0,25	≤0,125	8	≤4	≤1	≤1	≤0,0625	≤0,5	≤0,5	≤0,5	≤1/19	≤16	32
NT 6.5	≤2	≤2/2	≤4	≤4/4	≤1	≤1	≤0,5	≤0,5	≤0,25/4	≤0,5	≤2	≤4	0,5	≤0,25	≤0,125	≤4	≤4	≤1	≤1	≤0,0625	≤0,5	≤0,5	≤0,5	≤1/19	≤16	≤16
NT 9.1	≤2	≤2/2	≤4	≤4/4	≤1	≤1	≤0,5	≤0,5	≤0,25/4	≤0,5	≤2	≤4	0,5	≤0,25	≤0,125	8	≤4	≤1	≤1	≤0,0625	≤0,5	≤0,5	≤0,5	≤1/19	≤16	32
NT 9.2	≤2	≤2/2	≤4	≤4/4	≤1	≤1	≤0,5	≤0,5	≤0,25/4	≤0,5	≤2	≤4	0,5	≤0,25	≤0,125	8	≤4	≤1	≤1	≤0,0625	≤0,5	≤0,5	≤0,5	≤1/19	≤16	≤16
NT 9.3	≤2	≤2/2	≤4	≤4/4	≤1	≤1	≤0,5	≤0,5	≤0,25/4	≤0,5	≤2	≤4	0,5	≤0,25	≤0,125	8	≤4	≤1	1	≤0,0625	≤0,5	≤0,5	≤0,5	≤1/19	≤16	≤16
NT 9.4	≤2	≤2/2	≤4	≤4/4	≤1	≤1	≤0,5	≤0,5	≤0,25/4	≤0,5	≤2	≤4	0,5	≤0,25	≤0,125	≤4	≤4	≤1	≤1	≤0,0625	≤0,5	≤0,5	≤0,5	≤1/19	≤16	32
NT 10.1	≤2	≤2/2	≤4	≤4/4	≤1	≤1	≤0,5	≤0,5	≤0,25/4	≤0,5	≤2	≤4	0,5	≤0,25	≤0,125	8	≤4	≤1	≤1	≤0,0625	≤0,5	≤0,5	1	≤1/19	≤16	32
NT 10.2	≤2	≤2/2	≤4	≤4/4	≤1	≤1	≤0,5	≤0,5	≤0,25/4	≤0,5	≤2	≤4	0,5	≤0,25	≤0,125	8	≤4	≤1	≤1	≤0,0625	≤0,5	≤0,5	≤0,5	≤1/19	≤16	32
NT 11.1	≤2	≤2/2	≤4	≤4/4	≤1	≤1	≤0,5	≤0,5	≤0,25/4	≤0,5	≤2	≤4	0,5	≤0,25	≤0,125	≤4	≤4	≤1	≤1	≤0,0625	≤0,5	≤0,5	≤0,5	≤1/19	≤16	≤16
NT 11.8	≤2	≤2/2	≤4	≤4/4	≤1	≤1	≤0,5	≤0,5	≤0,25/4	≤0,5	≤2	≤4	0,5	≤0,25	≤0,125	≤4	≤4	≤1	≤1	≤0,0625	≤0,5	≤0,5	≤0,5	≤1/19	≤16	32
NT 13.2	≤2	≤2/2	≤4	≤4/4	≤1	≤1	≤0,5	≤0,5	≤0,25/4	≤0,5	≤2	≤4	0,5	≤0,25	≤0,125	16	≤4	≤1	≤1	≤0,0625	≤0,5	≤0,5	≤0,5	≤1/19	≤16	32
NT 18.4	>8	>32/2	≤4	≤4/4	≤1	≤1	≤0,5	≤0,5	≤0,25/4	≤0,5	>8	>16	1	≤0,25	≤0,125	16	≤4	≤1	≤1	≤0,0625	≤0,5	≤0,5	1	≤1/19	64	32
ZL 1.3	>8	>32/2	≤4	≤4/4	≤1	≤1	≤0,5	≤0,5	≤0,25/4	≤0,5	8	16	0,5	≤0,25	≤0,125	8	≤4	≤1	≤1	≤0,0625	≤0,5	≤0,5	≤0,5	≤1/19	≤16	32
ZL 2.5	≤2	≤2/2	≤4	≤4/4	≤1	≤1	≤0,5	≤0,5	≤0,25/4	≤0,5	≤2	≤4	0,5	≤0,25	≤0,125	8	≤4	≤1	≤1	≤0,0625	≤0,5	≤0,5	≤0,5	≤1/19	≤16	32
CA 3.4	≤2	≤2/2	≤4	≤4/4	≤1	≤1	≤0,5	≤0,5	≤0,25/4	≤0,5	≤2	≤4	≤0,25	≤0,25	≤0,125	≤4	≤4	≤1	≤1	≤0,0625	≤0,5	≤0,5	≤0,5	≤1/19	≤16	≤16

CA 9.6a	≤2	≤2/2	≤4	≤4/4	≤1	≤1	≤0,5	≤0,5	≤0,25/4	≤0,5	4	≤4	0,5	≤0,25	≤0,125	8	≤4	≤1	≤1	≤0,0625	≤0,5	≤0,5	≤0,5	≤1/19	≤16	32
CA 10.5	≤2	>32/2	≤4	≤4/4	≤1	≤1	2	≤0,5	≤0,25/4	≤0,5	4	16	0,5	≤0,25	≤0,25	8	≤4	≤1	2	≤0,0625	≤0,5	≤0,5	≤0,5	≤1/19	≤16	≤16
CA 10.6	≤2	≤2/2	≤4	≤4/4	≤1	≤1	≤0,5	≤0,5	≤0,25/4	≤0,5	4	≤4	0,5	≤0,25	≤0,125	8	≤4	≤1	2	≤0,0625	≤0,5	≤0,5	1	≤1/19	≤16	32
II CA 3.1	≤2	≤2/2	≤4	≤4/4	≤1	≤1	≤0,5	≤0,5	≤0,25/4	≤0,5	≤2	≤4	≤0,25	≤0,25	≤0,125	≤4	≤4	≤1	≤1	≤0,0625	≤0,5	≤0,5	≤0,5	≤1/19	≤16	≤16
II CA 3.6	≤2	≤2/2	≤4	≤4/4	≤1	≤1	≤0,5	≤0,5	≤0,25/4	≤0,5	≤2	≤4	≤0,25	≤0,25	≤0,125	≤4	≤4	≤1	≤1	≤0,0625	≤0,5	≤0,5	≤0,5	≤1/19	≤16	≤16
ED 1.1	≤2	≤2/2	≤4	≤4/4	≤1	≤1	≤0,5	≤0,5	≤0,25/4	≤0,5	≤2	≤4	0,5	≤0,25	≤0,125	8	≤4	≤1	2	≤0,0625	≤0,5	≤0,5	1	≤1/19	≤16	≤16
ED 1.3	≤2	≤2/2	≤4	≤4/4	≤1	≤1	≤0,5	≤0,5	≤0,25/4	≤0,5	≤2	8	≤0,25	≤0,25	≤0,125	8	≤4	≤1	≤1	≤0,0625	≤0,5	≤0,5	1	≤1/19	≤16	32
ED 1.4	≤2	≤2/2	≤4	≤4/4	≤1	≤1	≤0,5	≤0,5	≤0,25/4	≤0,5	≤2	≤4	0,5	≤0,25	≤0,125	8	≤4	≤1	≤1	≤0,0625	≤0,5	≤0,5	≤0,5	≤1/19	≤16	32
ED 2.3	≤2	≤2/2	≤4	≤4/4	≤1	≤1	≤0,5	≤0,5	≤0,25/4	≤0,5	≤2	≤4	1	≤0,25	≤0,125	8	≤4	≤1	≤1	≤0,0625	≤0,5	≤0,5	1	≤1/19	≤16	32
ED 3.1	≤2	≤2/2	≤4	≤4/4	≤1	≤1	≤0,5	≤0,5	≤0,25/4	≤0,5	≤2	≤4	0,5	≤0,25	≤0,125	8	≤4	≤1	2	≤0,0625	≤0,5	≤0,5	1	≤1/19	≤16	32
ED 3.2	≤2	≤2/2	≤4	≤4/4	≤1	≤1	≤0,5	≤0,5	≤0,25/4	≤0,5	≤2	8	1	≤0,25	≤0,125	8	≤4	≤1	2	≤0,0625	≤0,5	≤0,5	1	≤1/19	≤16	32

* white boxes indicate susceptibility, gray boxes indicate resistance to antimicrobials; AMP – ampicillin; AMC – amoxicillin/clavulanic acid; PIP – piperacillin, TZP piperacillin/tazobactam; AZE – aztreonam; FEP – cefepime; CFM – cefixime; CAZ – ceftazidime; CZA – ceftazidime/avibactam; CRO – ceftriaxone; CXM – cefuroxime; CF – cephalexin; IMP – imipenem; ERT – ertapenem; MEM – meropenem; TMO – temocillin; AK – amikacin; GEN – gentamicin; TOB – tobramicin; CIP – ciprofloxacin; LEV – levofloxacin; C – colistin; TGC – tigecycline; SXT – trimetoprim/sulfamethoxazole; FOS - fosfomicin; NF – nitrofurantoin.
